# The Formation of the Patterns of Desert Shrub Communities on the Western Ordos Plateau, China: The Roles of Seed Dispersal and Sand Burial

**DOI:** 10.1371/journal.pone.0069970

**Published:** 2013-07-26

**Authors:** Yange Wang, Xiaohui Yang, Zhongjie Shi

**Affiliations:** Institute of Desertification Studies, Chinese Academy of Forestry, Beijing, China; York U, Canada

## Abstract

The western Ordos Plateau is a key area of shrub diversity and a National Nature Reserve of endangered shrub species in north-west China. Desert expansion is becoming the most important threat to these endangered species. However, little is known about the effects of sand burial on the dynamics of the shrub community. This study aims to investigate how the shrubs as a community and as different individual shrubs respond to the disturbances caused by the desert expansion. The approach used by this study is to separate the seed-dispersal strategy from the sand-burial forces that are involved in structuring the shrub communities at different disturbance stages. Four communities for different disturbance stages were surveyed by using 50×50 m plots. The individual shrubs were classified into coloniser and successor groups at the seed-dispersal stage and strong and weak sand-burial tolerance groups at the sand-expansion stage. We employed spatial point pattern analysis with null models for each community to examine the seed-dispersal strategy and sand-burial forces affecting community distribution patterns. At the seed-dispersal stage, the interactions between the colonisers and the successors showed significant positive correlation at a scale of 0–1 m and significant negative correlation at a scale of 2 m; significant negative correlations between the groups with strong and weak sand-burial tolerance in the early stage of sand expansion at scales of 3–6 m, and significant positive correlation in the later stage of sand expansion at a scale of 13 m, were found. Seed-dispersal strategy is a reasonable mechanism to explain the shrub community pattern formation in the earlier stages, whereas sand burial is the primary reason for the disappearance of shrubs with weak sand-burial tolerance, this irreversible disturbance causes homogenisation of the community structure and produces aging populations of shrub species. This has an important influence on the succession direction of desert shrub communities.

## Introduction

The ecosystems of arid areas are among those in which plant facilitation is most frequently studied [1]–[4], especially in the seedling growth stage. In this stage, the ‘nursing’ of seedlings by shrubs is a pivotal feature of the shrub community [5]–[9]. Evidence from other arid ecosystems shows that in the community-formation stage, small-seeded species colonise bare ground, whereas large, wind-blown seeds are trapped by and become established under these existing small-seeded species initially [10]. Additionally, it is often assumed that low nutrient availability, harsh conditions and, in particular, low water availability are the predominant abiotic forces structuring plant communities in desert ecosystems [11]–[12]. Relatively few studies have assessed the impacts of biotic and abiotic forces on the abundance and distribution of shrubs and the community dynamics at different disturbance stages [13].

The western Ordos Plateau is the distribution center for northwest China's endemic genera and the key area of shrub diversity in northern China. More than 100 of the shrub and sub-shrub species found in arid and semi-arid areas of China are found in this area [14]–[16]. It is also a central area for endangered shrub species [17]. A National Nature Reserve focused on shrub protection has been established in the area. Meanwhile, human activities in the Ordos over the last 30 years have led to an expansion of the desert – arguably greater than the natural amount of expansion that occurred over the previous 2000 years. Desert expansion is becoming the most important threat to endangered shrub species [17]. Owing to irrational land reclamation, mining and grazing, a large area of fixed sand has changed to shifting sand. Driven eastward by the wind, the sandy areas have expanded. The shrub communities on the western Ordos Plateau have been affected by vegetation degradation and soil desertification over a long period [18]. However, little is known about how sand burial affects the dynamics of the shrub community.

This study aims to investigate how shrub-communities and different individual shrubs respond to desert expansion during escalating sand disturbances by separating the seed-dispersal stages from the sand-burial stages that eventually consume this ecosystem. We hypothesised that seed dispersal would determine shrub establishment success and that sand burial would affect plant health and growth, with important consequences for community structure and dynamics. Shrubs may be divided into two categories as coloniser and successor, according to their thousand-seed kernel weights (Table 1), which can be used to reflect the seed-disperal strategy [19]. Shrubs may also fall into two functional groups with respect to sand burial according to the sand thickness in the area in which their distribution is concentrated. We identified these two groups as the strong sand-burial tolerance group and the weak sand-burial tolerance group. We compared the abundance of the shrub species and the functional groups at each stage of disturbance, and we described the spatial distribution pattern of the members of the same or different groups at different scales to gain an understanding of these ecological systems and the dynamics of arid shrub communities.

**Table 1:** Classification of shrubs and classification criteria.

Species	Seed		Sand burial tolerance
	Thousand-seed kernel weight	Classification	
*Ammopiptanthus mongolicus* (Maxim.) S. H.Cheng	>10 g	successor	Strong
*Convolvulus fruticosus* Pall.	<10 g	colonizer	Weak
*Nitraria tangutorum* Bobrov.	>10 g	successor	Strong
*Potaninia mongolica* Maxim.	<10 g	colonizer	Weak
*Reaumuria songarica* (Pall.) Maxim.	<10 g	colonizer	Weak
*Reaumuria trigyna* Maxim.	<10 g	colonizer	Weak
*Sarcozygium xanthoxylon* Bunge	>10 g	successor	Weak
*Tetraena mongolica* Maxim.	<10 g	colonizer	Weak

(Ma, 1989; Zeng et al., 2000; Ma et al., 2003; Zhou et al., 2006).

## Materials and Methods

### Study Site and Species

**Figure 1:**
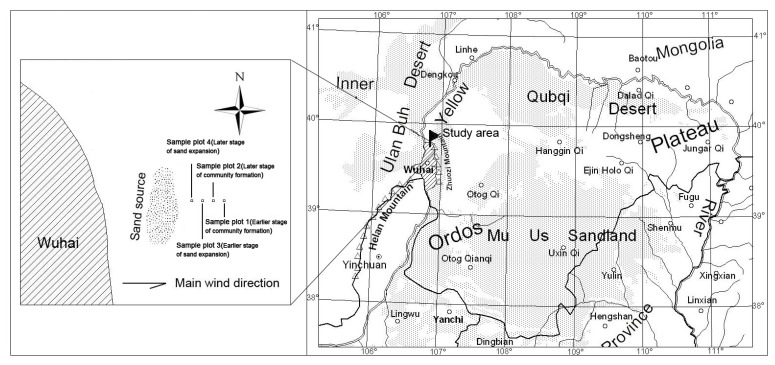
Map of Ordos Plateau and study region.

The study region is located in the western part of the Ordos Plateau, east of the Yellow River. It is administered from Wuhai City (106° 36′ – 107° 08′ E, 39° 13′ – 39° 54′ N, 1,150 m a.s.l.), Inner Mongolia. The Yellow River flows from south to north on the piedmont plain formed between the Zhuozi Mountains to the east and the Helan Mountains and Ulan Buh Desert to the west (Figure 1). The local climate is arid and temperate, with an annual precipitation of 150–200 mm, more than 60% of which occurs in July and August. The annual evaporation is 2,470.5–3,481 mm, the annual mean temperature is 7.8–8.1°C, the annual mean relative humidity is 42%, the annual mean wind speed is 2.7 m s^−1^, the sand-blown period is 41–67 days, and the total annual amount of solar radiation is 155.9 kcal cm^−2^. The main soil types are grey desert soil, aeolian soil, and meadow soil, with pH values of 9.0–10.0. Wuhai is located in an ecotone ranging from desert steppe to steppe desert. The average vegetation cover is 25%, and the desert shrubs and semi-shrubs are *Tetraena mongolica* Maxim. and *Ammopiptanthus mongolicus* (Maxim.) S. H. Cheng.

### Data Collection

Experiments to study pattern-generating processes in arid and semi-arid plant communities, such as the shrub communities investigated here, are difficult. These communities are characterized by slowly changing dynamics and by a disturbance process defined over long time scales because of the difficulty of seed germination and the presence of species that exhibit relatively slow growth and long life spans [20]. Therefore, this study used the spatial distribution of shrubs as a proxy for the progress of time to identify the drivers and trends of disturbance.

In a preliminary survey, we found that the shrub communities surveyed were located in the leeward region of a large sand-source area to the east of the city of Wuhai. This area is expanding toward the east. The sand source was a sand mound devoid of vegetation. Most of the plant species that we studied are wind dispersed, whereas few are gravity dispersed. Because both seed dispersal and sand transmission are primarily wind driven, both the disturbance stages and the intensity of sand burial as well as seed dispersal should follow gradients along the wind direction. In August 2006, we selected four typical sample plots as the representative of a gradient to represent different disturbance stages of the shrub communities along the main wind direction. Sample plot 1 was located at a distance of 2,000 m from the sand source on the west slope of a low hill (Figure 1) covered by gravel at the eastern edge of the concentrated shrub distribution area (furthest from the sand source). This plot was considered to represent an earlier stage of community pattern formation. Sample plot 2 was located at a distance of 1,500 m from the sand source and was covered by gravel and a few shallow patches of sand. It was considered to represent a later stage of community pattern formation. Sample plot 3 was located at a distance of 1,000 m from the sand source. It was covered by a 5-cm deep layer of sand and was considered to represent an earlier stage of sand expansion. Sample plot 4 was located 500 m from the sand source and was covered by sand approximately 12 cm deep. This plot was considered to represent a later stage of sand expansion. Both the shrub density and the proportion of seedlings decreased systematically from east to west (sample plot 1 to sample plot 4). Many sand dunes were present around the shrubs in sample plot 4. Some of the dunes reached 1.5 m high and 3 m wide along the wind direction. The plots had not been disturbed by humans or grazed by livestock over the 15 years preceding the study. Rare natural granivores and herbivores were found in the area.

**Figure 2:**
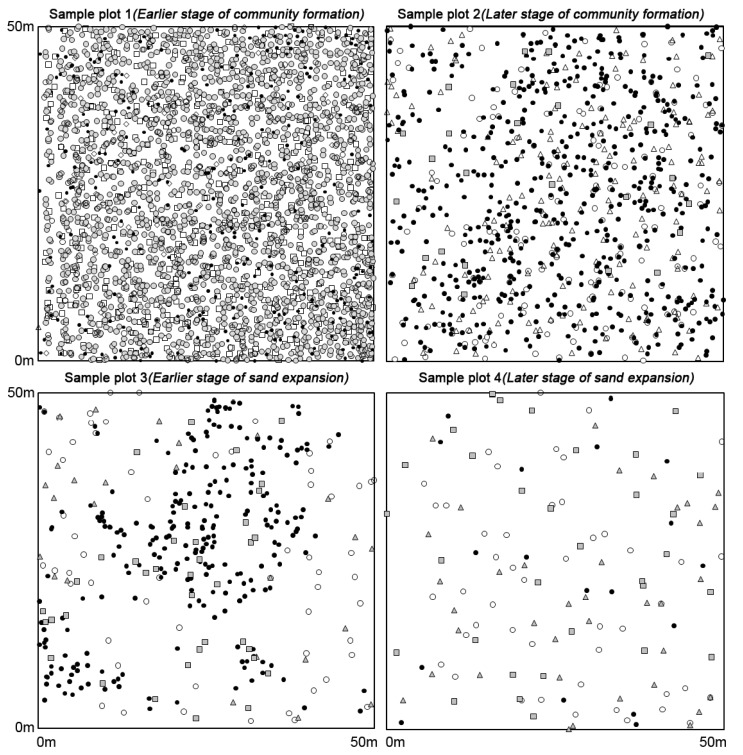
Point map of shrubs.

One 50 m×50 m subplot was established for each of the four sample plots, which is suitable size to document shrub diversity (see species-area curve by Yang [21]). All individual shrubs in the four subplots were mapped using a total station transit (model GTS-3B, Topcon, Paramus, New Jersey) and classified into different groups according to their biological, physiological and ecological characteristics (Table 1, Figure 2). Because some shrubs are capable of clonal reproduction, we considered the ramets as distinct individuals if they grew more than 10 cm apart.

### Spatial Statistics

Ripley’s K-function and the pair-correlation g-function are common techniques for univariate and bivariate point-pattern analysis [22]–[23]. They are used together by ecologists because they have different sensitivities. In some situations, it is difficult to precisely reflect the pattern of certain scale features using Ripley’s K-function because of the cumulative ‘memory’. However, the g-function, which has been developed from Ripley’s K-function and uses rings instead of circles, allows specific distance classes to be isolated so that it can detect aggregation or dispersion accurately at a given distance, *t* [24]. The g-function can also reflect spatial pattern types more effectively [25]. The g-function has been shown to be preferable to the commonly used Ripley’s K-function in research similar to the current study [19]. Consequently, in this study we only used the g-function in spatial point pattern analysis.

The univariate g-function is determined by Equation 1 [24]:
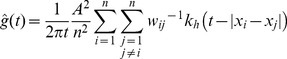
(1)Where *A* is the plot area, *n* is the total number of plants and *w_ij_* is a weighting factor correcting for edge effects. *k_h_* is a kernel function – a weight function applying maximum weight to point pairs with a distance exactly equal to *t* but incorporating point pairs at an approximate distance *t* with reduced weight. This weight falls to zero if the actual distance between the points differs from *t* more than *h*, the so-called bandwidth parameter.

Values of 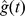
>1 indicate that inter-point distances of *t* are more frequent and that samples are aggregated, whereas values of 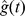
<1 indicate that they are less frequent and that samples are dispersed compared to spatial randomness.

The bivariate point patterns concern the spatial distribution of the individuals of one species in relation to those of another species. The corresponding bivariate spatial point pattern is estimated by
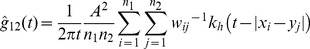
(2)Where *x_i_*, *i* = 1, …, *n_1_*, and *y_j_*, *j* = 1,…, *n_2_* are the points of groups 1 and 2, respectively, with the same weights *w_ij_* and kernel function *k_h_* as Equation 1.

### Univariate and Bivariate Null Models

The homogeneity test showed that the colonisers and successors as well as all of the shrubs in sample plot 1 were homogeneously distributed, whereas on sample plots 2, 3, and 4, heterogeneity was found for each functional group and for all the shrubs.

We applied the complete spatial randomness model (CSR) [26] to sample plot 1 because the CSR is the simplest and most widely used null model for homogeneous patterns. For sample plots 2, 3 and 4, we applied the heterogeneous Poisson process null model, the simplest alternative to CSR for heterogeneous patterns [27].

We applied an antecedent-condition null model to test the hypothesis that small-seeded species colonise bare ground and that large, wind-blown seeds are trapped by and become established under the existing small-seeded species [10] for the communities on sample plot 1 and sample plot 2. This null model is commonly used when two types of points are created in sequence rather than at the same time. We kept the location of the colonisers fixed and randomised the successors according to the CSR model and the heterogeneous Poisson process in simulations for sample plots 1 and 2, respectively [27].

To test the sand-burial hypothesis on sample plots 3 and 4, we applied a random-labelling null model. The model was used to investigate whether the labels ‘weak sand-burial tolerance’ and ‘strong sand-burial tolerance’ have a random structure within the given spatial structure of the joint pattern [27].

We calculated the 99% Monte Carlo confidence envelopes for each null model by running 99 Monte Carlo simulations. The Programita software, written by Wiegand and Moloney [25], was used for the calculations.

## Results

### Changes of Species in Different Sand-Burial Gradients

In sample plot 1, which represents the early stages of community pattern formation, four out of the five species were colonisers. The abundance of *Tetraena mongolica*, a weak sand-burial tolerant coloniser, reached a peak in sample plot 2 and then decreased gradually after sand burial. The abundance of *Nitraria tangutorum*, a strong sand-burial tolerant successor, reached its peak in sample plot 3 and then decreased. Shrubs with weak sand-burial tolerance disappeared gradually during the sand expansion stage. Plot 4 primarily included strongly sand-burial tolerant shrubs. The total number of shrubs decreased gradually from 2,893 to 135 during the process of disturbance produced by the sand expansion (Figure 2, Table 2).

**Table 2:** Shrub numbers in four sample plots.

Species	Sample plot 1	Sample plot 2	Sample plot 3	Sample plot 4
*Ammopiptanthus mongolicus*			25	37
*Convolvulus fruticosus*	135			
*Nitraria tangutorum*		25	117	32
*Potaninia mongolica*	1 784			
*Reaumuria songarica*		164		
*Reaumuria trigyna*	494			
*Sarcozygium xanthoxylon*	209	162	59	48
*Tetraena mongolica*	271	555	246	18

### Spatial Point Pattern Analysis Based on the Hypothesis of Seed Dispersal Strategy

**Figure 3:**
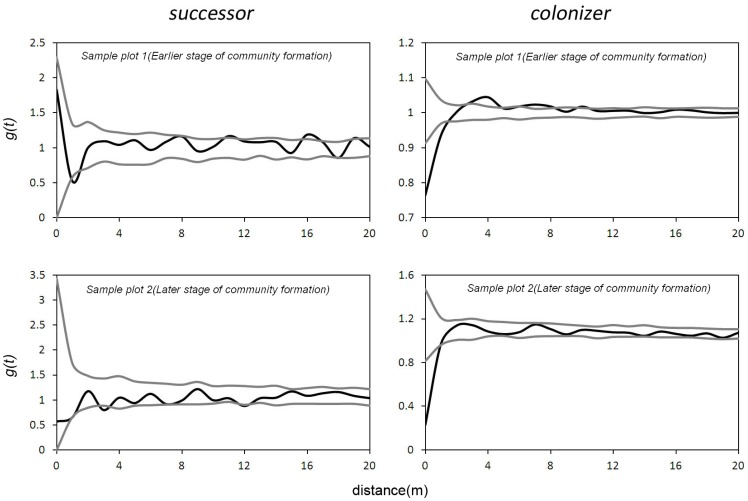
Univariate g(t) function of colonizers and successors in the community formation stage.

Based on the seed dispersal strategy, we classified the shrubs into colonisers and successors. The spatial point pattern analysis showed that in the early stage of community pattern formation (sample plot 1), the colonisers had a uniform distribution at a scale of 0–1 m and an aggregated distribution at scales of 3–4 m. The successor *Sarcozygium xanthoxylon* had a uniform distribution at scales of 1 m and 18 m and an aggregated distribution at a scale of 11 m. In the later stage of community pattern formation (sample plot 2), the colonisers had a uniform distribution at a scale of 0–1 m, whereas the successors *Nitraria tangutorum* and *Sarcozygium xanthoxylon* had a uniform distribution at scales of 0–1 m, 3 m and 12 m (Figure 3).

**Figure 4:**
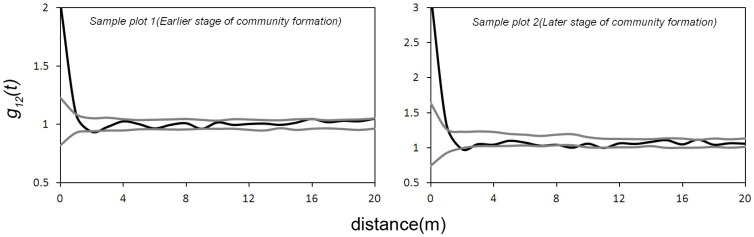
Bivariate g12(t) function between colonizers and successors in the community formation stage.

The interactions between the colonisers and the successors showed a significant positive correlation at a scale of 0–1 m and a significant negative correlation at a scale of 2 m in sample plot 1 and plot 2 (Figure 4).

### Spatial Point Pattern Analysis Based on the Hypothesis of Sand Burial Effects

**Figure 5:**
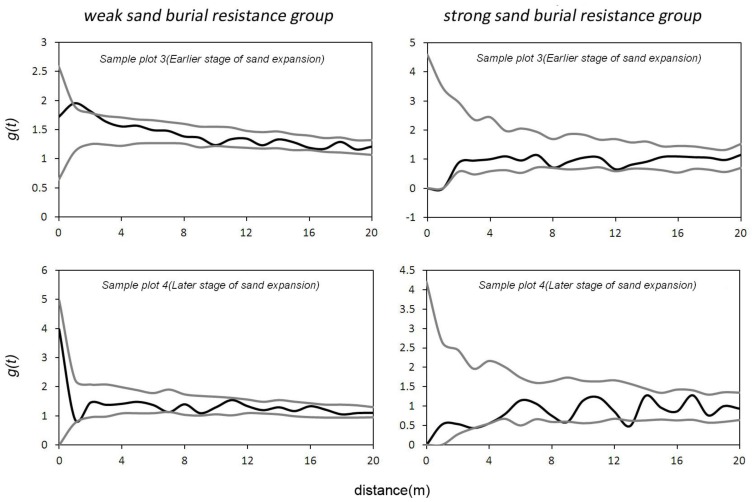
Univariate g(t) function of strong and weak sand burial resistance groups in the sand burial stage.

In the early stage of sand expansion (sample plot 3), the weak sand-burial tolerance group had an aggregated distribution at a scale of 1–2 m, and the strong sand-burial tolerance group had a uniform distribution at a scale of 7 m (Figure 5). In the later stage of sand expansion (sample plot 4), the weak sand-burial tolerance group had a random distribution at all scales that we studied, and the strong sand- burial tolerance group had a uniform distribution at the scales of 9 m and 13 m (Figure 5).

**Figure 6:**
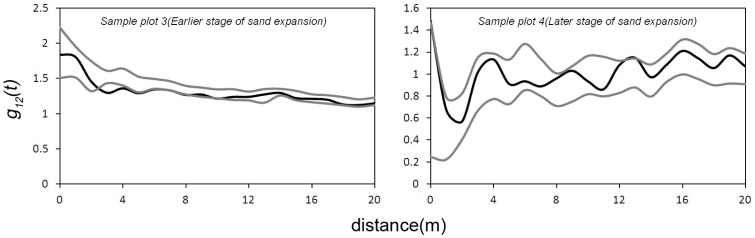
Bivariate g12(t) function between strong and weak sand burial tolerance groups in the sand burial stage.

Significant negative correlations were found between the groups with strong and weak sand-burial tolerance in the early stage of sand expansion (sample plot 3) at scales of 3–6 m, whereas in the later stage of sand expansion (sample plot 4) a significant positive correlation was found at a scale of 13 m (Figure 6).

## Discussion

### Effect of Sand Burial on Community Succession

Sand burial, as an important natural selection pressure of plant distribution in desert areas, play an important role on the community succession [28]–[29]. Some studies showed that sand burial can enhance the seed germination and seedling emergence of the sand-burial tolerant shrubs [30]–[31] (Zhang et al., 2010; Li and Zhao, 2006). During the process of disturbance in the western Ordos Plateau shrub communities, small-seeded shrubs play a dominant role in the early stage of community development, whereas strongly sand-burial tolerant shrubs play a dominant role after sand invasion. The total number of individuals in the shrub communities decreased gradually as the sand invaded. We assumed that each sample plot corresponded to a different stage of community disturbance. During disturbance, as successors invade, the occurrence of the colonisers is gradually reduced and the colonisers may even disappear. In the first disturbance stage, the only successor was *Sarcozygium xanthoxylon*; in the second disturbance stage, the coloniser *Reaumuria songarica* and the successor *Nitraria tangutorum* had invaded, whereas the colonisers *Reaumuria trigyna, Convolvulus fruticosus* and *Potaninia mongolica* had disappeared; and in the third and fourth stages, as a result of sand burial, the only remaining shrub species were the strongly sand-burial tolerant shrubs *Nitraria tangutorum* and *Ammopiptanthus mongolicus* and the weakly sand-burial tolerant shrubs *Sarcozygium xanthoxylon* and *Tetraena mongolica*. The thick sand covering was only suitable for the seed germination of a few large-seeded and strongly sand-burial tolerant shrubs that have a stronger emergence capability [32]. Consequently, few seedlings occurred in the sand-burial communities, and the community composition tended to be simplified and tended to include older-aged shrubs. Thus, following sand invasion, the abundant shrubs and the high diversity of the shrub communities in study area, even on the whole western Ordos Plateau [16], gradually declined.

### Small-Scale Aggregated Distributions in the Early Stage of Community Pattern Formation

In the early stages of community pattern formation, the colonisers had an aggregated distribution at a scale of 3–4 m, which may result from accumulation of small seeds in the lowlands by wind transport when they invade the communities [33]–[36]. However, both sample plots 1 and 2 showed a uniform distribution on a scale of less than 1 m. This pattern was probably a result of shrub competition [19]. Moreover, in these two stages all the coloniser shrubs had their own crown sizes but were represented by points in the statistical analysis. This simplification may obscure an aggregated distribution at small scales [37]. The successors were distributed almost randomly in the early stage of community pattern formation. This result indicates that large seeds do not accumulate easily in one place.

The bivariate spatial point pattern analysis indicated that a significant positive correlation occurred between the coloniser and successor groups in the early stages of community pattern formation. This result indicates that the seeds of the successors may be trapped by the colonisers that may facilitate the establishment of successors there [10].

The univariate and bivariate pattern analysis above showed that the colonisers had an aggregated distribution at small scales in the early stage of community pattern formation. Such a pattern may occur because shrubs with small seeds, such as *P. mongolica*, colonise bare ground first [10] by accumulating in small depressions, such as those formed by wind [33]–[34], [38]–[39]. Both colonisers and successors (for example, *N. tangutorum*) may co-occur at a small scale in the early stage because new seeds can be trapped by the existing colonisers and become established there [1], [40]. Other factors causing small-scale aggregated distribution may include soil heterogeneity resulting from the presence of micro-environments in which seedlings can grow under adult plants, the so-called ‘nurse-plant syndrome’ [40]–[44]. In these situations, adult plants alter the environment. For example, the surface temperatures in the open space between shrubs and under the canopy are different, and the adult plants may regulate the temperature [1], [45]–[47]. The seed-dispersal strategy appears to represent a reasonable mechanism for explaining the dominant shrub community distribution pattern that occurs during the early stages of disturbance. This explanation is similar to the conclusions of a previous analysis of spatial patterns in a semi-arid Karoo shrubland [19].

### Sand-Burial Effects

Plants encounter different levels of sand burial in sand dune habitats [48]–[50]. Sand burial can change the physiology, morphology, growth, and survival of plants by altering the biotic and abiotic environment [51]–[53] through parameters such as available photosynthetically active radiation [54]–[55], humidity, and temperature [29], [56]. Different species have different degrees of sand-burial tolerance, and sand burial, as an environmental sieve, has driven the development of sand-burial tolerance mechanisms over the course of evolution [51]. Only those species with a certain degree of tolerance to sand burial can survive and grow in areas with drifting sand.

Moderate sand burial can improve the environment by increasing the humidity around plant roots and promoting the activities of soil microorganisms. Shrub species with root-suckering ability are prone to grow adventitious roots and continuously increase in size buried by sand [57]–[58]. In turn, this process increases the height of the plant buried by sand, promoting its biomass accumulation and the formation of a new branch [54], [59]. However, sand burial gradually changes from a positive effect to a negative effect if the intensity of the sand burial is greater than a certain threshold. Plants cannot photosynthesise in the dark following sand burial [54]. This process weakens plant growth and can affect plant survival [48], [51], [56]. Sand burial results in the formation of a large, high dune around the bush, changing the horizontal distribution of soil material [60]–[61]. Nutrients and moisture within the communities can be displaced horizontally, causing marked differences between the soil on the sand dunes and in deflation hollows between the dunes and thereby affecting the distribution patterns of plants.

At the beginning of sand accumulation, the weak sand-burial tolerance group had an aggregated distribution at small scales. This distribution may have resulted from the primary reproductive mode of weakly sand-burial tolerant shrubs changing to sprouting from seed propagation and by small shrubs growing around the maternal shrub [16]. At the later stage of sand burial, only a few weakly sand-burial tolerant maternal shrubs were left in the community with random distribution, while most of seedlings or juvenile shrubs with weak tolerance around them disappeared. The number of strongly sand-burial tolerant shrubs also decreased under sand-burial pressure, compared with the earlier stage. They were randomly distributed at most of the scales studied and showed a uniform distribution at only a few scales (i.e. 9 m and 13 m).

Species with strong and weak sand-burial tolerance exhibited a significant negative correlation at the early stage of sand-burial expansion (sample plot 3). A thick layer of sand covered the ground, and sand dunes formed around the shrubs with strong sand-burial tolerance. As a result, these shrubs had few of the weakly sand-burial tolerant shrubs in their vicinity. At the later stage of sand burial, weakly sand-burial tolerant species tended to become extinct, and the number of strongly sand-burial tolerant species also decreased. The correlation between the groups was weak or even absent because shrubs were sparsely distributed in the community.

Sand burial and wind erosion are two important sources of selection pressure for plant distributions in arid areas [62] and are also the most common source of perturbation? in grasslands, desertified grasslands, and desert areas in China [63]. These two factors play important roles in the formation of plant communities in habitats with drifting sand [48] and represent important driving forces of community disturbance [64]. Our analysis shows that sand burial may cuase the disappearance of species with weak sand-burial tolerance which are dominant species in undegraded shrub communities. Sand burial has an important influence on the direction of development in shrub communities on the western Ordos Plateau and is also an important cause of decreasing shrub diversity in this region. Following the decline and death of the shrubs, large numbers of sand dunes become active and mobile. This change initiates a vicious cycle that affects all downwind areas.

In this paper, we hypothesized two primary driving forces for shrub-community spatial patterns in different disturbance stages on the western Ordos Plateau. Spatial analyses showed that the patterns of shrub communities may be formed by the seed-dispersal strategy. However, this mechanism breaks down in the later sand-expansion stage because seed germination is difficult in the changed environment. Sand burial seems to be the main force driving community degradation. This process causes the species with weak sand-burial tolerance to disappear. The disturbance is irreversible, causes homogenisation of the community structure and produces ageing populations of shrub species. We should be aware that this study is a preliminary attempt using a typical community investigation, which may over- or underestimate influence of the two primary driving forces on the western Ordos Plateau shrub pattern formation, and using spatial pattern models, which can only reflect underlying ecological processes with apparent patterns, hence, further works should put emphasis on more experimental studies of more shrub communities in this region in order to have deeper insight into underlying ecological processes of shrub community pattern formation.
